# Oral cancer awareness campaign in Northern Germany: successful steps to raise awareness for early detection

**DOI:** 10.1007/s00432-023-04820-0

**Published:** 2023-05-03

**Authors:** Eva Baumann, Michael Koller, Hans-Jürgen Wenz, Jörg Wiltfang, Katrin Hertrampf

**Affiliations:** 1 Department of Journalism and Communication Research, Hannover University of Music, Drama, and Media, Germany, Expo Plaza 12, 30539 Hannover, Germany; 2 Centre for Clinical Studies, University Hospital Regensburg, 93042 Regensburg, Germany; 3 Department of Prosthodontics, Propedeutics and Dental Materials, University Hospital Schleswig-Holstein, Campus Kiel, Arnold-Heller Str. 3, Building B, 24105 Kiel, Germany; 4 Department of Oral and Maxillofacial Surgery, University Hospital Schleswig-Holstein, Campus Kiel, Arnold-Heller Str. 3, Building B, 24105 Kiel, Germany

**Keywords:** Oral cancer, Socio-economic status, Cancer awareness campaign, Early detection

## Abstract

**Purpose:**

Oral cancer is an underestimated health problem, and its existence and the relevant prevention measures are not sufficiently known by the general population. The project thus aimed to develop, implement and evaluate an oral cancer campaign in Northern Germany, and to increase problem awareness on various levels: draw public attention to the tumour by media coverage increase awareness of early detection opportunities for the target group, and raise awareness of carrying out early detection measures by the professional groups involved.

**Methods:**

For each level, a campaign concept was developed and documented in terms of content and timing. The identified target group was elderly educationally disadvantaged male citizens ≥ 50 years. The evaluation concept for each level included pre-, post- and process evaluations.

**Results:**

The campaign was carried out from April 2012 to December 2014. The issue of awareness within the target group was significantly increased. Media coverage showed that regional media adopted the topic of oral cancer and placed it on their published agenda. Furthermore, the continuous involvement of the professional groups over the course of the campaign led to an increased awareness of oral cancer.

**Conclusion:**

The development of the campaign concept with a comprehensive evaluation showed that the target group was successfully reached. The campaign was adapted to the required target group and specific conditions, and was also designed to be context sensitive. It is, therefore, recommended that the development and implementation of an oral cancer campaign on a national level be discussed.

**Supplementary Information:**

The online version contains supplementary material available at 10.1007/s00432-023-04820-0.

## Introduction

Oral and pharyngeal cancers are still among the most underestimated cancers (oral cavity including lips and pharyngeal region; ICD-10 C00-C14) and, against this background, poses a particular challenge in regard to public health promotion. In Germany, the number of new cases of this tumour entity shows an increasing trend of almost 14,000 cases diagnosed annually. Relative to other cancer sites, it was seventh in men (3.8% of all cancer cases) and 15th in women. The five-year survival rates were 47% and 63%, respectively. With a median age at onset of 63 years for men and 66 years for women, the majority of people affected are older (Robert Koch Institut 2021). In contrast to stagnant high incidences in men, an increasing trend has been observed in women in recent years. More than 50% of all diagnosed cases for both sexes were localised exclusively in the oral cavity (Hertrampf et al. 2015; Jansen et al. 2021).

Although the standards of treatment for oral cancer from a dental or medical perspective for diagnosis and therapy have improved in recent decades, this has not had a positive impact on early detection. One reason for this is that two-thirds of those affected do not consult a dentist, oral or maxillofacial surgeon, or other specialist until the tumour is at an advanced stage (Joseph 2002; Laronde et al. 2008; Mignogna et al. 2001; Patton et al. 2004). Earlier diagnosis would improve the likelihood of survival and could reduce treatment-related limitations for patients (Cheung et al. 2021).

However, earlier diagnosis presupposes knowledge about the disease and existing possibilities for early detection. A minimum level of awareness of the risk or problem and attention to the topic should be brought to the notice of the general population. However, a number of international studies, as well as our own results, showed that the population has insufficient knowledge about oral cancer, its risk factors, symptoms and possible preventive measures, specifically for early detection (Hertrampf et al. 2012; Horowitz et al. 2002; Patton et al. 2004; West et al. 2006). Beyond the general population's insufficient level of knowledge, these studies were able to identify a lower level of knowledge among subjects 60 years and older and/or with a lower level of schooling (Hertrampf et al. 2012; Patton et al. 2004; Tomar and Logan 2005). With regard to issue awareness, a separate study showed that this was significantly lower among men than women, and that educational differences were also evident here (Baumann et al. 2016).

In Germany, in contrast to other tumour entities for which education and screening programmes have already been carried out, such as for skin cancer (Geller et al. 2010; Greinert et al. 2003) or colorectal cancer (Loss et al. 2006), there have been no activities in the past to inform risk groups comprehensively and in a structured way about oral cancer and the possibilities for prevention and early detection. The routine visual inspection of the oral mucosa is the most important diagnostic measure for making an initial tentative diagnosis. This examination is painless for the patient, is not time-consuming and has no side effects. In Germany, it is integrated into the six-monthly or annual routine dental examination required by the health system and covered by health insurance.

A prerequisite for successful awareness campaigns is a communication strategy that is developed on a theoretical basis and systematically evaluated in all phases (Valente 2001). This raises the question of the requirements for a scientifically based awareness campaign. An intervention strategy to promote prevention and health promotion should address the individual behavioural determinants and contextual factors relevant to the specific health behaviour in a communicative way, and contribute to a change in attitude and behaviour (Bartholomew and Mullen 2011; Michie et al. 2008).

Studies from the USA and Great Britain were able to show that campaigns of different durations with a theory-based, systematic structure led to an improvement in awareness, as well as problem and topic perception regarding oral cancer (Eadie et al. 2009; Jedele and Ismail 2010; Watson et al. 2009). However, successful prevention strategies need to be planned and designed in a context-sensitive way (Sallis et al. 2008). The transferability of internationally successful strategies is, therefore, only possible to a limited extent, i.e. concept development is always necessary where the intervention strategy is not only adapted to specific target groups and socio-structural factors, but must also take into account national and regional economic, socio-political and environmental conditions, as well as the national healthcare system.

Therefore, the aim was to raise awareness for oral cancer in people at increased risk as the main target group. By using the federal state of Schleswig–Holstein as a model region, we asked for the practicability and effectiveness of a systematic multi-level approach in campaign development and evaluation.

## Materials and methods

### The awareness campaign

Based on a comprehensive conceptual framework (Baumann et al. 2019a, b), a campaign was developed and implemented in the federal state of Schleswig–Holstein with the primary aim of publicising the existence of this tumour and raising awareness of oral cancer. This is derived from the transtheoretical model of behaviour change (Prochaska and DiClemente 1983), according to which a sustainable change in health behaviour—i.e. the development of prevention behaviour—requires an increase in problem awareness at the first stage of intention formation.

The campaign was designed to increase problem awareness on various levels:

- Media coverage mass media level: draw public attention to the existence of the tumour, prevention options, and campaign activities.
- Target group level (risk group): increase awareness of the topic and the problem, with the aim of perceiving early detection opportunities.
- Level of the professional groups involved: raising awareness of the issues and problems, with the aim of carrying out early detection measures.
- Epidemiological level: temporary increase in incidence and diagnosis at an earlier tumour stage; generating supplementary data to identify the target group (already published (Hertrampf et al. 2020).

The campaign was launched in the third week of April 2012 and kicked off with a press conference, running until December 2014. The content and timing of the campaign was laid down in a project schedule, and the use of each medium and event was documented as Gantt-Figure (supplementary information). In order to reach the high-risk group—elderly and educationally disadvantaged male citizens with social and health problems—a regional network of the City Mission, consumer centres and health offices were also established, and contacts were made with facilities for the homeless and welfare associations. Through the welfare organisations, the railway station missions, lunch counters, church socials, addiction clinics, debt counselling, senior citizens' facilities, community centres and outpatient care were also involved. All these institutions supported the campaign on a voluntary basis.

The project was approved by the Ethics Committee of the Medical Faculty of the Christian Albrecht University (A113/06).

### The evaluation concept

The relevance of a comprehensive evaluation strategy is also evident for the prevention of oral cancer: existing studies indicate that a formative, in-process and summative evaluation is useful and necessary to enable specific target group planning, implementation and effectiveness measurement points for the campaign (Eadie et al. 2009; Jedele and Ismail 2010; Logan et al. 2013; Watson et al. 2009). The four levels of the campaign objectives guided the evaluation concept.

For the levels of the public media, the risk group as a target group within the population and for the professional groups involved are specifically developed and elaborated on below. By combining these different concepts, an evaluation matrix was created for the entire campaign. Along this matrix, the requirements for all evaluation areas were defined and implemented (Baumann et al. 2019a, b). The methodology and its results described below refer to process evaluation and summative evaluation. In the sense of a process analysis, the process evaluation aimed to record possible fluctuations and developments (e.g. in public awareness, in the level of knowledge, in the perception of problems, in the beginning of possible changes in behaviour), and to be able to react to them. Indications of external influences can also be identified in this way. At the same time, the process evaluation provided possible explanations for the results of the summative evaluation, which aims to measure the effectiveness of the campaign after its completion.

### Public relations

The extent to which the mass media run with a topic is an important indicator of public perception and awareness of that topic. This increases the topic’s relevance among the general population. At the same time, medical journalistic reporting is a relevant source of information for citizens when it comes to risks, possibilities of prevention and early detection or therapies (Niederdeppe et al. 2008), also with regard to the topic of cancer (Niederdeppe et al. 2007; Rutten et al. 2006).

In the context of public relations for the campaign described above, the work done in the media had the primary goal of increasing public awareness of the issue through using journalists as gatekeepers, and initiating reporting on the campaign. Numerous measures were implemented to achieve this. The choice of communication tools and channels was adapted to the risk and high-risk group within their specific social and cultural environment, and to the area of campaign distribution. In addition, the media presence was increased through setting related measures and involving multipliers. The media mix comprised earned media (press coverage, including local, regional daily and weekly press, television and radio in the campaign area, as well as thematically relevant media such as health insurance magazines and health magazines), owned media (website, flyers, mouth model as a three-dimensional information and experience space) and paid media (posters in public transport, advertising on shopping carts) (Yadav and Kobayashi 2015).

**Fig. 1:**
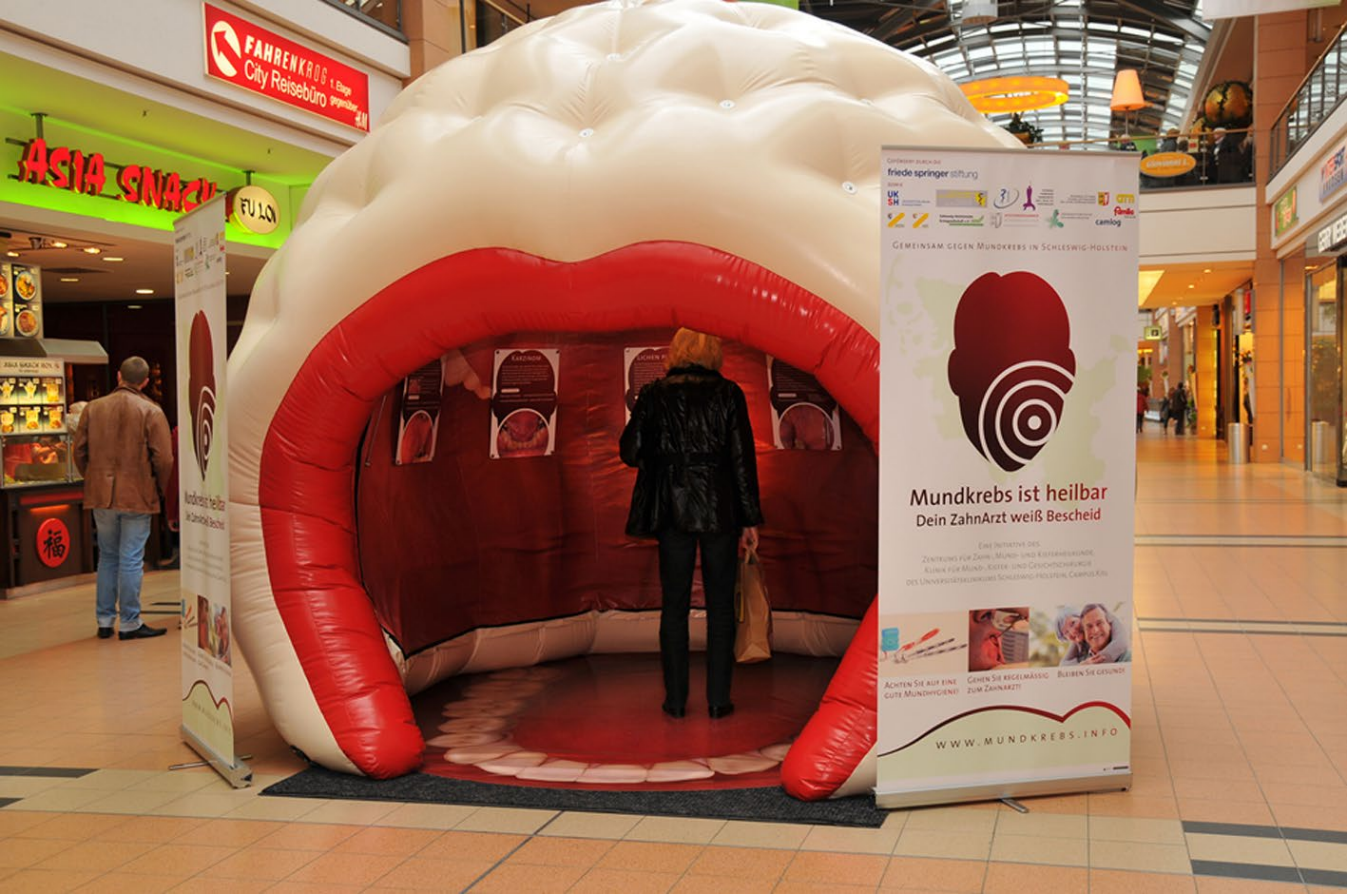
The walk-in mouth model

For the campaign’s media and public relations work, a concept was developed in advance to initiate media-compatible and newsworthy reporting occasions during the actual campaign. In preparation, press contacts were researched, coaching for the project leaders on public relations and dealing with journalists was realised, and a media campaign (logo and slogan, website, flyers, posters), as well as a walk-in mouth model, were developed (Fig. 1). Against the background of the barriers and uncertainties identified in the risk and high-risk group in dealing with the topic (Baumann et al. 2019a, b), the campaign messages were designed accordingly in terms of form and content. This resulted in, among other things, the use of the more easily understandable term "mouth cancer" instead of "tumour of the oral cavity", and a positive, preferably not fear-fuelling address for all communication. Instead of a portrait photo, which can cause identification barriers with the person shown, a neutral, stylised representation was chosen as the visual key in the media and in the logo (Baumann et al. 2019a, b).

Contacts were established with journalists from the local and regional press in the areas of print (daily newspapers and health media, as well as member magazines, e.g. of health insurance companies), radio and TV, and maintained throughout the campaign. A press conference was organised for the campaign launch, interviews with journalists from the local and regional press, and appearances at events (e.g. sporting events) or a presence at consumer fairs were organised. The entire press work was comprehensively documented.

In addition to establishing contact with journalists, contact was made with other stakeholders who acted as multipliers, especially for the high-risk group, by distributing the media campaign (posters, flyers) free of charge and providing display areas (e.g. social institutions, welfare organisations, consumer advice centres, shopping centres). Self-initiated and on-demand realised mailing campaigns were also documented.

By December 2014, the walk-in inflatable mouth model had been presented a total of 27 times at 12 different locations, such as shopping malls, with each campaign being accompanied by press work (press releases, interviews). The presentation lasted between one day and 3 weeks per location. The average was one to two weeks.

The constant exchange between those responsible for the project and the press representatives aimed at maintaining the presence of the topic, meeting the information needs of journalists, and acting as contacts for the topic. The direct feedback from the journalists, the organisers and the local citizens served to adjust the measures and align them with the questions, interests and information needs of the people. If there was interest, further information material was always made available to lay people as well as to the media representatives.

For monitoring media awareness and evaluating the media work of the campaign, a standardised content analysis of the reporting on oral cancer was carried out. This was started before the campaign began in order to create a reference value for the type and intensity of reporting after the campaign had started, and—for the first year of the campaign—to be able to trace the changes in media attention during the process, and to measure the success or response to the media work, as well as to individual campaign activities.

We conducted a content analysis of articles on oral cancer in print newspapers. Since the campaign took place in Northern Germany, we selected articles from northern local (e.g. Schleswig-Holsteinischer Zeitungsverlag, Hamburger Abendblatt) and national (e.g. Frankfurter Allgemeine Zeitung, Süddeutsche Zeitung) daily newspapers that were published between January 2012 and August 2013. The articles were derived from the German media monitoring service “PMG Pressemonitor” using a previously developed and tested search term (“mouth” AND “cancer” OR “tumour” OR “ulcer” etc.) for a systematic full-text search, which was kept constant for the entire survey period and daily query.

A standardised codebook was developed as a survey instrument, on the basis of which all articles previously identified as relevant were coded in an identical manner, and which enabled a systematic description of media coverage that is comparable across all articles. In addition to formal characteristics of the article (e.g. date of publication, medium, length of the text), content-related reporting characteristics (e.g. main topic of the article, significance of the topic of oral cancer in the article) were also collected (this will not be discussed further with regard to general media attention and the media response to the campaign, which is the focus here). Five coders were trained to code all articles that at least mentioned the topic “oral cancer”. Holsti's intercoder reliability (Holsti 1969) ranging from 0.82 to 1.0, indicates acceptable to very good scores.

### General public and risk group

For the evaluation of the campaign in the general population, a quantitative representative computer-assisted telephone survey (*n* = 500) was conducted at several measurement points with the risk group—50 years and older—in the campaign’s distribution area, i.e. with residents in Schleswig–Holstein, using a revised and modified questionnaire based on the 2007/2008 survey for target group identification (Hertrampf et al. 2012):

- Baseline (t0) (March 2012)
- Start of prevention campaign (April 2012)
- 1st process evaluation (t1) November 2012
- 2nd process evaluation (t2) November 2013
- After completion of campaign (t3) November 2014.

The representative sample was drawn after a systematic random selection, based on an ADM telephone sample (ADM = Working Group of German Market and Social Research Institutes), and was realised as a computer-assisted telephone interview (CATI method) by a polling firm. The questionnaire covered various topics (e.g. level of knowledge, perception of topics and problems, health indicators, perceptions and experiences of interaction with the dentist and oral mucosal examination, health information behaviour, campaign contact), whereby only selected parameters are presented for the evaluations reported here.

Descriptive statistics of respondents’ demographic characteristics and survey responses were reported by means of counts and percentages. Survey responses were reported for the total sample (*n* = 500) and broken down by the following demographic and socio-economic criteria: gender, age, employment, and graduation. We calculated the difference in percentages of correct answers per item between each demographic segment (e.g. men, aged 50–59) and the total sample of *n* = 500. The statistical significance level was *α* = 5%; *p*-values < 0.05 were considered statistically significant. All analyses were computed with SPSS software (SPSS inc., Chicago, Illinois, USA).

### Dental and other medical professional groups

As part of the development of the campaign concept, a uniform concept for the involvement of the respective professional groups in the campaign was developed with the regional dental association, the regional medical association, and the professional associations for otorhinolaryngologists, dermatologists and general practitioners.

Prior to the start of the campaign, the respective professional groups within the federal state were first informed in writing about the commencement of the campaign in a joint letter from the associations and professional associations, as well as the project management, and they were given the opportunity to order specific campaign materials free of charge in the form of posters and flyers.

After 12 months, they were written to again (in 2013 and 2014), and the campaign to order posters and flyers free of charge was repeated. Orders could be placed by fax, post or email. The responses and the amount of material ordered were documented. The documentation was done according to the occupational group and number of orders. Personal data was not collected.

During the course of the campaign, it was also documented whether this concept of repeated addressing was accepted by the different professional groups, and whether more colleagues in private practice became sensitised to the topic of “oral cancer”.

#### Inclusion criteria

Reported practices of dentists, oral surgeons, oral and maxillofacial surgeons, otorhinolaryngologists, dermatologists and general practitioners within Schleswig–Holstein (data from the Schleswig–Holstein Dental and Medical Association):

- Dentists, oral surgeons, maxillofacial surgeons: 1500 practices = 1800 persons
- otorhinolaryngologists: 98 practices = 123 persons
- Dermatologists: 107 practices = 126 persons
- General practitioners: 1800 persons

(Data on practices and persons describe the average over the project period.)

The number of orders by professional group was recorded anonymously in an Excel spreadsheet. The evaluation was purely descriptive according to absolute and relative frequencies.

## Results

In the course of the campaign, no events occurred that fundamentally changed the campaign implementation process and need to be considered in the summative evaluation at any of the different evaluation levels.

### Media coverage

**Fig. 2:**
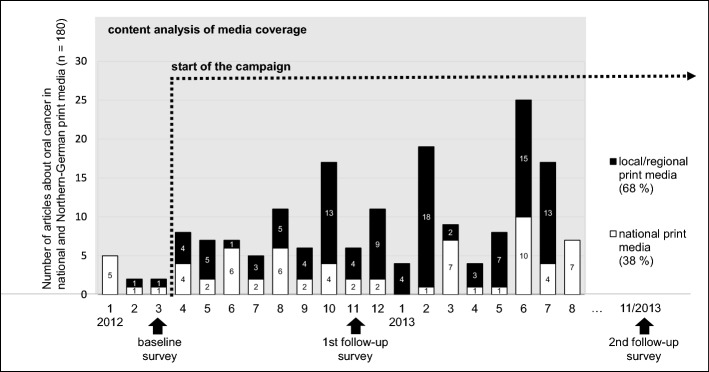
Description of content analysis of media coverage

**Fig. 3:**
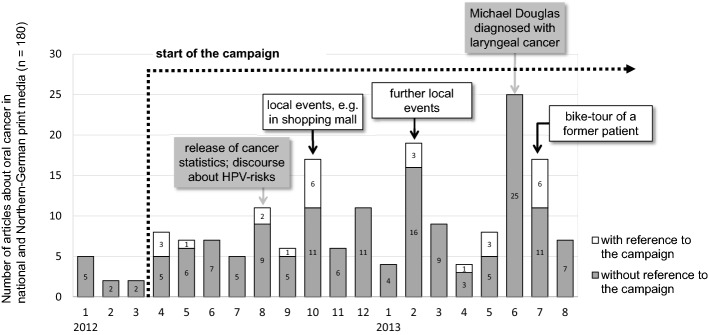
Description of media analysis with and without reference to the campaign

Firstly, a frequency analysis shows how the presence of oral cancer in the media changed after the campaign started (Fig. 2). Even though we can only compare the monthly coverage for 3 months before the campaign started, it seems clear that oral cancer was a notable media topic more often during the campaign period than before it. It also shows that media coverage is subject to fluctuations that may be influenced by campaign activities, by external events related to the topic, or by competing topics (Fig. 3).

In view of the regional campaign focus on the federal state of Schleswig–Holstein and the campaign activities realised here, it is useful to differentiate to what extent the local/regional press differs from the national press. As displayed in Fig. 2, we can see that the topic was highlighted particularly by the regional daily print media. We can cautiously draw the conclusion that the local and regional media in Schleswig–Holstein has been prompted to put oral cancer on the agenda.

In addition, the media content analysis provides information on the extent to which the campaign was referred to in press coverage (media resonance analysis).

Here we can see that the media did react to and refer to the campaign. Overall, 14% (*n* = 26) of the articles published on oral cancer in any thematic context contained explicit references to the campaign. It is clear that some media coverage was initiated by campaign activities, for example, by the exhibition of an artificial model of the oral cavity and the mouth that was displayed in shopping malls, or a bike tour of a former oral cancer patient who acted as an advocate for the campaign. Additional reporting was inspired by other stimuli such as public health reports, science communication efforts or traditional news factors like stories about celebrities.

### General public and risk group

The primary goal of the campaign was to evaluate the effects, especially on the attention and perception of oral cancer, over time. Increased behavioural intention or even behavioural change always requires attention and perception of the “problem”, and is thus always difficult to achieve through a pure communication campaign. In this context, the results on attention and perception of “mouth cancer” are crucial and will be focussed on here (Table 1).

**Table 1:** Description of the evaluation on the awareness of the topic “mouth cancer” across all measurement points for the total cohort and stratified according to socio-economic factors

Awareness of the issue “mouth cancer”	Baseline March 2012	1st process evaluation November 2012	2nd process evaluation November 2013	Post-campaign November 2014
Total cohort (*p* < 0.000) (*n* = 500 vs. *n* = 500 vs. *n* = 499 vs. *n* = 499)	25.4% (*n* = 127)	39.8% (*n* = 199)	39.1% (*n* = 195)	43.3% (*n* = 216)
Sex				
Men (*p* = 0.056) (*n* = 232 vs. *n* = 232 vs. *n* = 232 vs. *n* = 231)	23.7% (*n* = 55)	32.8% (*n* = 76)	31.5% (*n* = 73)	34.6% (*n* = 80)
Women (*p* < 0.000) (*n* = 268 vs. *n* = 268 vs. *n* = 267 vs. *n* = 268)	26.9% (*n* = 72)	45.9% (*n* = 123)	46.1% (*n* = 123)	50.7% (*n* = 136)
Age groups				
50–59 years (*p* = 0.008) (*n* = 163 vs. *n* = 164 vs. *n* = 164 vs. *n* = 164)	22.7% (*n* = 37)	32.3% (*n* = 53)	36.0% (*n* = 59)	39.6% (*n* = 65)
60–69 years (*p* = 0.013) (*n* = 146 vs. *n* = 145 vs. *n* = 145 vs. *n* = 145)	29.5% (*n* = 43)	46.9% (*n* = 68)	43.4% (*n* = 63)	43.4% (*n* = 63)
70–79 years (*p* = 0.002) (*n* = 151 vs. * n* = 145 vs. *n* = 153 vs.* n* = 150)	24.5% (*n* = 37)	41.4% (*n* = 60)	39.2% (*n* = 60)	44.7% (*n* = 67)
> 80 years (*p* = 0.074) (*n* = 38 vs. *n* = 45 vs. *n* = 38 vs. *n* = 40)	23.7% (*n* = 9)	40.0% (*n* = 18)	36.8% (*n* = 14)	52.5% (*n* = 21)
Education				
Elementary school (*p* = 0.010) (*n* = 115 vs. *n* = 122 vs. *n* = 98 vs. *n* = 104)	22.6% (*n* = 26)	41.8% (*n* = 51)	39.8% (*n* = 39)	36.5% (*n* = 38)
Secondary school (*p* < 0.001) (*n* = 172 vs. *n* = 179 vs. *n* = 201 vs. *n* = 176)	26.7% (*n* = 46)	38.5% (*n* = 69)	41.3% (*n* = 83)	48.3% (*n* = 85)
Baccalaureate (*p* = 0.002) (*n* = 207 vs. *n* = 190 vs. *n* = 194 vs. *n* = 213)	26.1% (*n* = 54)	40.5% (*n* = 77)	38.1% (*n* = 74)	42.7% (*n* = 91)

For mouth cancer awareness, there was a positive effect across all measurement points for the total cohort (representative sample of people aged 50 and over in Schleswig–Holstein). Almost a quarter (25.4%, *n* = 127) of respondents in the baseline survey vs. 39.8% (*n* = 199) in the first process evaluation after seven months and respectively, one year later—November 2013 (39.1%, n = 195) and in the final evaluation in November 2014 (43.3%, *n* = 216)—stated to have heard or read about mouth cancer. This increased awareness and attention was significant (+ 17.9%, *p* < 0.000).^1^

Stratification by socio-demographic and socio-economic factors showed a significant increase in perception for both genders. The increase for women was + 23.8% (from *n* = 72 to *n* = 136) (*p* < 0.000), and for men + 10.9% (from *n* = 55 to *n* = 80) (*p* = 0.056), which contributed to an increasing gender gap over time, as women began at an already higher level.

The age comparison showed that in all age groups there was a clear increase in topic awareness of at least 14% between the baseline and the final evaluation. While there was a continuous positive trend of + 16.9% (from *n* = 37 to *n* = 65) (*p* = 0.008) among those aged 50–59, attention to the topic among those aged 60–69 (+ 13.9%, from *n* = 43 to *n* = 63, *p* = 0.013), as well as among those aged 70–79 (+ 20.2%, from *n* = 37 to *n* = 67, *p* = 0.002), increased significantly at the beginning and remained stable at this high level. The group of 80-year-olds and older showed a clear improvement in absolute terms, but was not significant given the small number of cases (+ 29.2%, *n* = 9 to *n* = 21, *p* = 0.074).

Stratification according to educational background showed a positive trend for the perception of and attention to the topic of mouth cancer across all measurement points in all educational backgrounds. This increased most clearly in the group with secondary school diplomas (+ 21.6%, *n* = 46 to *n* = 85, *p* = 0.001), followed by the group with lower secondary school diplomas (+ 13.9%, from *n* = 26 to *n* = 38, *p* = 0.010) and the group with high school diplomas (+ 16.6%, *n* = 54 to *n* = 91, *p* = 0.002) from 13.9% to 21.6%. The detailed presentation is given in Table 1.

Further evaluations on uncertainties and fears in dealing with the topic of cancer, and specifically with the topic of oral cancer, as well as on attitudes towards prevention options during the course of the campaign are planned for a further publication.

### Dental and other medical professional groups and further stakeholders

In cooperation with the regional dental and medical associations, and the respective medical professional associations, the relevant professional groups were personally informed about the campaign and continuously involved. Before the campaign started, the number of dental practices, practices for maxillofacial surgery, oral surgery and otorhinolaryngologists were very similar (Table 2). Among otorhinolaryngologists, almost the same proportion (22%) reported ordering information material for the first time in spring 2013. Among dentists, oral and maxillofacial surgeons, 13% reported ordering for the first time. The increase in new practices was lower after the renewed contact in spring 2014, but overall, 45% of all dentists, oral surgeons and maxillofacial surgeons in Schleswig–Holstein actively registered at least once during the campaign and ordered information material. Among otorhinolaryngologists, about half of all practices in the state reported ordering at least once. Among dermatologists, significantly fewer practices reported overall and the increase was the same, but overall participation was significantly lower compared to other professional groups.

**Table 2:** Description of the response rate from the practices at the different measurement points due to material order

	Material order March 2012	Material order Spring 2013	Material order Spring 2014
Dentists, oral surgeons, maxillofacial surgeons (*n* = 1500)	22.00% (*n* = 329)	35.00% (+ 13%, *n* = 195*)	45.00% (+ 10%, *n* = 153*)
Otolaryngologist (*n* = 98)	21.00% (*n* = 45)	43.00% (+ 22%, *n* = 49*)	51.0% (+ 8%, *n* = 27*)
Dermatologists (*n* = 107)	8.00% (*n* = 9)	11.00% (+ 3%, *n* = 3*)	14.00% (+ 3%, *n* = 3*)

*The + sign refers to the practices that reported for the first time

Since the assignment to the respective occupational group was made via the practice stamp, it was not possible to clearly identify practices with general practitioner (GP) training. Therefore, no data could be generated in this regard. Against this background, this occupational group is not listed in Table 2. Overall, the response for practices from the area of internists and GPs was less than 5% after the last contact in spring 2014. For all professional groups, the majority of contacts to the working group were made by fax. In total, only 65 practices across all occupational groups could not be traced over all three contacts due to unidentifiable addresses.

Other stakeholders, such as the association of pharmacies and the regional health offices, were also personally informed about the campaign and continuously involved. This personal contact has proven to be an effective multiplier function. The project schedule (Gantt-Figure supplementary information) shows how impressively this fast-ball effect has been established and documents the importance of intensive networking.

A total of 130,525 flyers and 3325 posters were sent out by December 2014. Flyers and posters were sent to pharmacies (73,000 flyers, 2190 posters) and health offices (750 flyers, 90 posters) as an unsolicited “circular”. All other flyers and posters were actively requested by the institutions and partners to support the campaign via email, fax or post.

## Discussion

The described prevention campaign to improve early detection of oral cancer is the first campaign in Germany that developed and implemented a regional media-based communication strategy for this topic, and also conducted a comprehensive ex-ante [formative] and ex-post [summative] evaluation, including process evaluation (Bonfadelli and Friemel 2020).

The focus of this campaign was on issue and problem awareness (information about the existence of the tumour), and the associated increase in awareness within the risk and high-risk group (Bonfadelli and Friemel 2020). This primary goal of making known the existence of the disease “mouth cancer” was successfully implemented.

One reason for improved awareness and attention in the target group, as also shown by international studies, is due to the systematic and theory-based structure (Eadie et al. 2009; Jedele and Ismail 2010; Watson et al. 2009). This concept of differentiation into different thematic levels allowed for an adequate description and evaluation of each sub-area, but also enabled a well-founded assessment regarding the achievement of objectives for the entire campaign.

It can also be stated that the concept developed in advance to increase or sensitise the media (public) in the sense of health media agenda building has proven successful (Martinson and Hindman 2005). Before the campaign, there was very little media coverage of the disease. The discussions with the various—mainly local and regional—media representatives on the relevance of the topic, and the question of whether and how it could be presented more prominently, clearly increased the relevance from the journalists’ point of view. In addition, it appears that it was possible for them to set up related actions through their own activities in order to increase visibility in the media. This concept enabled continuous reporting on the disease. An important aspect that contributed to continuous reporting was the regular exchange with the press representatives. This made it possible to take up suggestions and tips from the press representatives and to align the press work with the needs of the journalists (Fromm et al. 2011). It resulted in the topic of oral cancer being successfully covered in the media, and that it was also possible to raise awareness of prevention challenges.

Another important aspect of the campaign and public relations work was the distribution of information material, free of charge, by the large number of multipliers from the various sectors. The setting-related measures and the integration of the multipliers in the course of a regional network formation have proved to be successful. Thus, the establishment of the regional network was accompanied by a continuous exchange, through which the presence of the topic could be maintained. In addition, new multipliers were continuously recruited during the course of the campaign. This work, both for the press network and for the network of multipliers, was very time-consuming and personnel-intensive in terms of development, continuous exchange and individual approaches.

Beyond the documentation of press contacts and press network activities, the media analysis showed that the media presence of the topic could be increased, which was not the case in international published studies (Canto et al. 1998; Graham et al. 2004).

The focus of this campaign was primarily to inform about the existence of the tumour (problem perception) within the identified target group (men, ≥ 50 years, educationally distant background). The results on target group outreach showed a significantly improved awareness by the end of the campaign. This speaks for an increased awareness of the topic or that the topic has reached the target group. These results on the increase in awareness and attention to the oral cavity tumour correspond to the campaign success of Watson et al. (2009) and Jedele and Ismail (2010), with a campaign duration of 12 and 24 months respectively (Jedele and Ismail 2010; Watson et al. 2009).

The results among the sexes, in the age group 60 years and older, as well as in the group of people with the lowest education, showed clearly positive effects with regard to attention and awareness. The increase in awareness in the age group 60 years and older is particularly important when seen against the background of this tumour mainly occurring in people older than 60 years (Robert Koch Institute 2021), and therefore educational work must specifically be carried out in this target group.

Furthermore, increased awareness among women should be positively highlighted, as women tend to have a stronger interest in health topics and are more active in seeking health information—not only for themselves but also for relatives (Galarce et al. 2011; Rutten et al. 2006). These important results regarding the accessibility of the identified target group was also shown by Watson et al. (2009) and Jedele and Ismail (2010) in their studies (Jedele and Ismail 2010; Watson et al. 2009).

The different, non-linear increase within the individual subgroups underlines the necessity of process evaluations. They enable a reflection of the campaign's orientation and a statement on how well which sub-target group could be reached, with which measure, and in which phase of the campaign. Based on these results, any necessary modifications can be made (Randolph and Viswanath 2004). Furthermore, process evaluations are necessary in order to be able to make statements about the awareness of the topic and of its relevance in the course of the campaign period (Atkin and Freimuth 2001).

Since oral cancer is not diagnosed on a daily basis in the day-to-day practice of dentists and medical disciplines, a campaign concept with regard to content and timing was coordinated with associations and professional associations for the continuous involvement of the various professional groups. This earliest possible involvement and coordination with associations and professional associations already proved effective in the formative evaluation (Hertrampf et al. 2014, 2011). The concept was intended to sensitise colleagues in private practice to the topic, but at the same time not to influence the decision as to which information materials would be used and how. Therefore, the decision was made in favour of a written announcement with an invitation to order, and against a generalised mailing of information material for all practices, which was also intended to avoid wastage. In addition, this made it possible to document the information materials requested, and the form of contact between dentists and doctors in private practice and the working group. This active request for information material showed an increase in topics and problem awareness.

### Limitations

A statement on the measures of effectiveness should always be discussed against the background of the overall results of the different sub-areas (media public, professional groups, target group). Nevertheless, a separate result should be formulated for each sub-area. For example, an increased awareness of the disease in the target group initiated by the campaign should not lead to the conclusion that a higher proportion of the target group also attends dental check-ups. These aspects must be taken into account so that the results are not over- or misinterpreted when interpreting the degree of achievement of the campaign goals (effectiveness). It must also be taken into account that external influences via media could have an impact on campaign results. However, these influences can be minimised by a comprehensive evaluation concept.

The presented prevention campaign with its evaluation concept was supported in its implementation by many voluntary institutions, organisations and multipliers. On the one hand, this has reduced monetary resources, but on the other hand it has made this area personnel- and time-intensive. Therefore, in a discussion to raise this concept to a national level, the experiences of national institutions should be used, especially for the context-sensitive concept development, as the aspect of voluntary support can only be transferred and realised on a national level to a limited extent, and also involves a high documentation effort.

## Conclusion

In summary, the concept development for the campaign with the comprehensive evaluation of the different sub-areas showed that the target group was successfully reached. The campaign was adapted to the required specific target group conditions and was also designed to be context-sensitive (Sallis et al. 2008). This made it possible to systematically generate results for the various sub-areas of this complex intervention with regard to accessibility. The awareness of issues and problems within the target group was significantly increased. Therefore, the working group recommends discussing the development and implementation of a campaign for oral cancer on a national level. This national concept should be oriented towards the regional concept and thus be based on the draft of an evaluation matrix with the described levels as a starting point. This offers the possibility of adapting the different sub-areas to the national situation and to modifying the necessary evaluation concepts.

## Supplementary Information

Below is the link to the electronic supplementary material.

Supplementary file1 (PDF 200 KB)

## Data Availability

All datasets generated or analysed during the current study are available from the corresponding author on reasonable request.

## Footnotes

1 The results of the baseline and first process evaluation on issue awareness have already been published (Baumann et al. 2016). However, in order to be able to present an effect for the entire campaign, it is imperative to present the results in their entirety and over time.
