# Supplementary material for: Oral cancer awareness campaign in Northern Germany: successful steps to raise awareness for early detection
Source: J Cancer Res Clin Oncol. 2023 May 3;149(11):8779–89. doi: 10.1007/s00432-023-04820-0 (PMC10374765; doi:10.1007/s00432-023-04820-0)
Supplement: Supplementary file 1 — Supplementary file1 (PDF 200 KB) [file 432_2023_4820_MOESM1_ESM.pdf]

	1st Quarter 2012			2nd Quarter 2012			3rd Quarter 2012			4th Quarter 2012			1st Quarter 2013			2nd Quarter 2013			3nd Quarter 2013			4th Quarter 2013			1st Quarter 2014			2nd Quarter 2014			3rd Quarter 2014			4th Quarter 2014		
	Jan.	Feb.	Mar.	Apr.	May	June	July	Aug.	Sep.	Oct.	Nov.	Dec.	Jan.	Feb.	Mar.	Apr.	May	June	July	Aug.	Sep.	Oct.	Nov.	Dec.	Jan.	Feb.	Mar.	Apr.	May	June	July	Aug.	Sep.	Oct.	Nov.	Dec.
**Mass media**																																				
Bus-Indoor posters Flensburg-Lübeck																																				
Bus-Indoor posters Kiel-Neumünster																																				
Indoor posters German railways, regional																																				
Posters on shopping trolleys in 30 supermarkets																																				
**Health-PR/Medical journalism**																																				
Telephone action "Citizens ask experts"																																				
TV feature, private channel																																				
TV feature North German Radio																																				
Radio feature North German Radio 1																																				
Press release of the project group on mouth model																																				
Newspaper article initiated by project group																																				
Magazine article on homeless people																																				
Article in campus magazine of the University Hospital																																				
Aricle in member magazine of Cost Unit																																				
New Pharmacy magazine article																																				
Lecture by the project group for lay people																																				
**Presentation "walk-in inflatable mouth model" and sporting events**																																				
Norla, largest regional agricultural exhibition																																				
Health exhibition (Neumünster)																																				
Shopping malls/town halls																																				
Shopping malls Kiel																																				
Shopping malls Lübeck																																				
THW Kiel (handball), Holstein Kiel (soccer)																																				
**Use of own channels**																																				
Own campaign website																																				
Linking to other websites																																				
**Subject-specific multipliers**																																				
Pharmaceutical association: Dispatch posters/flyers																																				
Article in regional pharmacist magazine																																				
Dental practices: Dispatch posters/flyers																																				
Article in regional dental journal																																				
Doctors practices: Dispatch posters/flyers																																				
Article in regional magazine SHI*																																				
Articel in regional medical magazine																																				
Article with the support of the GSDOM press office**																																				
**Multipliers within Schleswig-Holstein who have continuously requested information materials**																																				
Terminal Cruise Ferry Kiel-Oslo																																				
Regional Cancer Association																																				
Oncology Outpatient Clinic/City Hospital Kiel																																				
Occupational Medicine Kiel																																				
Holstein Kiel (soccer)																																				
Addiction counselling Kiel Horizon																																				
Social Church District of Gaarden-Kiel																																				
City missions																																				
Independent patient information																																				
Consumer centres incl. debt counselling																																				
Welfare organisations (AWO, Caritas, Diakonie)***																																				
State Association for Health Promotion																																				
Public Health Offices																																				
Town halls, District administrations (sporadically)																																				

Appendix: Project schedule with documentation of events and media over the course of the campaign (the preparatory phase is shown in light blue).

* Association of the Statutory Health Insurance Dentists of Schleswig-Holstein, **German Society for Dentistry and Oral Medicine,

***Includes AWO community centres and service houses; includes Caritas homeless people, debt counselling, health counselling, lunch counters, railway station missions; includes Diakonie homeless people, addiction clinics, debt counselling, outpatient care.
